# Integrated Approaches for the Management of Staple Line Leaks following Sleeve Gastrectomy

**DOI:** 10.1155/2017/4703236

**Published:** 2017-02-02

**Authors:** Mauro Montuori, Domenico Benavoli, Stefano D'Ugo, Luca Di Benedetto, Emanuela Bianciardi, Achille L. Gaspari, Paolo Gentileschi

**Affiliations:** ^1^Bariatric Surgery Unit, Department of Experimental Medicine and Surgery, University of Rome Tor Vergata, 00133 Rome, Italy; ^2^Psychiatry Unit, Department of Systems Medicine, University of Rome Tor Vergata, 00133 Rome, Italy

## Abstract

*Introduction*. Aim of the study was trying to draw a final flow chart for the management of gastric leaks after laparoscopic sleeve gastrectomy, based on the review of our cases over 10 years' experience.* Material and Methods*. We retrospectively reviewed all patients who underwent LSG as a primary operation at the Bariatric Unit of Tor Vergata University Hospital in Rome from 2007 to 2015.* Results*. Patients included in the study were 418. There were 6 staple line leaks (1.44%). All patients with diagnosis of a leak were initially discharged home in good clinical conditions and then returned to A&E because of the complication. The mean interval between surgery and readmission for leak was 13,4 days (range 6–34 days, SD ± 11.85). We recorded one death (16.67%) due to sepsis. The remaining five cases were successfully treated with a mean healing time of the gastric leak of 55,5 days (range 26–83 days; SD ± 25.44).* Conclusion*. Choosing the proper treatment depends on clinical stability and on the presence or not of collected abscess. Our treatment protocol showed being associated with low complication rate and minor discomfort to the patients, reducing the need for more invasive procedures.

## 1. Introduction

Higher morbidity and mortality in overweight and obesity have been observed for hypertension, diabetes mellitus, heart disease, dyslipidaemia, stroke, osteoarthritis, sleep apnoea, cancer, and more comorbidities [1]. Initially described as a first-stage procedure for the treatment of super-obese patients, laparoscopic sleeve gastrectomy (LSG) has rapidly spread for its indisputable advantages such as absence of implantable foreign body, avoidance of malabsorption, relative simplicity of execution, maintenance of gastrointestinal continuity and good results in terms of weight loss [2].

Despite technical improvements of surgical staplers and reinforcement materials, the rate of complications related to the staple line remains relevant, mainly because of bleeding and leaks.

In this study, we focused on staple line leaks, based on data collected and recorded on a prospective database. We describe here our experience over 10 years with the management of gastric leaks in a series of patients submitted to a primary LSG, trying to draw a final flow chart based on our findings.

## 2. Methods

We reviewed the notes of all patients who underwent LSG as a primary operation for morbid obesity at the Bariatric Unit of Tor Vergata University Hospital in Rome, from 2007 to 2015.

All patients were evaluated before surgery by a multidisciplinary team including surgeons, endocrinologists, psychiatrists, nutritionists, and anaesthesiologists.

Preoperative work-up included esophagogastroscopy, barium swallow, blood samples, chest X-ray, electrocardiogram, and when needed spirometry, echocardiography, and polysomnography.

Psychiatric counselling was conducted with the aim of excluding patients unsuitable for surgery due to mental health contraindications [3].

Demographic, comorbidity, surgical, weight, height, BMI, admission information, imaging tests, complications, and reoperations data were prospectively collected.

Second-generation cephalosporin was given for antibiotic prophylaxis.

All LSGs were performed by the same surgical team using a standard technique.

Under general anaesthesia, 5 trocars were inserted through the abdominal wall. The greater omentum was dissected away from the greater gastric curvature with the harmonic scalpel (Ultracision® Ethicon). A 36-French bougie (12 mm) was positioned in the stomach, close to the lesser curvature, as a calibration for the gastric resection, starting about 5 cm laterally to the pylorus up to the angle of His. Subsequential firings with Echelon® 60 mm staplers (Ethicon Endosurgery Cincinnati, OH) with blue and green reloads, reinforced in selected cases with either Peristrips-Dry® (Synovis, St. Paul, MN) or Seamguard® (W.L. Gore & Associates, Elkton, MD, USA) were performed, closed to the bougie [4].

An intraoperative staple line test with methylene blue was performed using the orogastric tube. The resected stomach was extracted from the abdomen and a closed suction drain was placed along the staple line.

At postoperative day (POD) 2, all patients underwent a radiological upper gastrointestinal series, and in absence of leakage they started a liquid diet and the drain was removed. Patients were usually discharged at POD 3 with nutritional indications. A week later, all patients returned to the outpatient clinic for the first follow-up appointment.

## 3. Results

From 2007 to 2015, 418 patients underwent LSG (159 males, 259 females). The mean BMI was 45.90 Kg/m^2^ (range 27.68–70.0 Kg/m^2^; SD ± 7.47); the mean operative time was 85.42 minutes (range 45–205 minutes; SD ± 32.42) and the mean length of stay was 3.55 days (range 2–14 days; SD ± 1.63).

All procedures were performed laparoscopically with no conversion to open surgery.

No major intraoperative complications were recorded and neither intraoperative nor perioperative deaths within 24 hours after surgery were noted.

There were 6 staple line leaks (1,44%), in 2 females and 4 males. They had a mean BMI of 45.44 kg/m^2^ (range 34.29–69.20 kg/m^2^; SD ± 13.79). The mean operative time was 93 minutes (range 60–170 minutes; SD ± 43.82) and the mean length of stay 3.8 days (range 3–5 days; SD ± 0.84).

The mean interval between surgery and readmission for clinical presentation of leak was 13.4 days (range 6–34 days, SD ± 11.85).

We record 1 acute leak (presentation 6 days after sleeve gastrectomy) and 5 early leaks (presentation 7–34 days after surgery).

No significant risk factor was found.

Clinical presentation varied between fever spike, tachycardia over 120 beats per minute, and abdominal heaviness up to septic shock.

In all cases the leaks were located at the gastrooesophageal junction area, along the suture line.

Only one of the patients with leak (16.67%) had staple line reinforcement during surgery.

**Figure 1:**
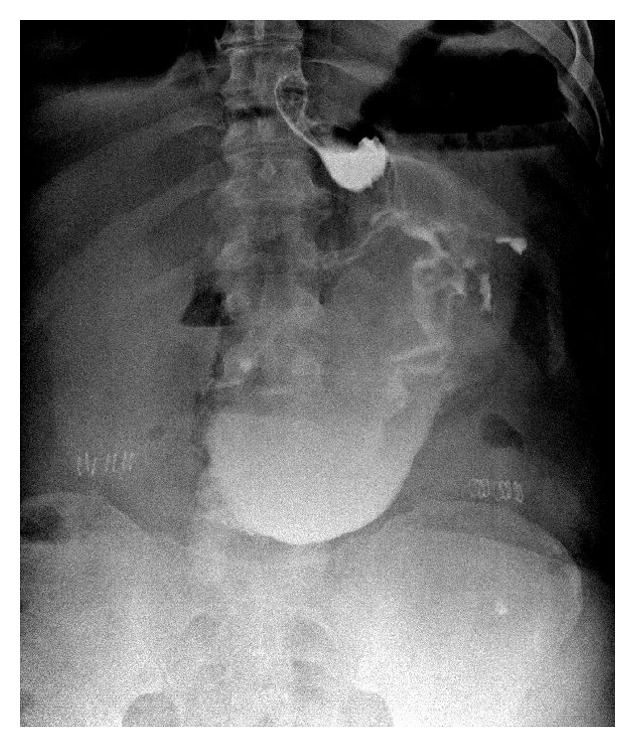
Male, 48 years, BMI 43 Kg/m^2^. Evidence of leakage at radiological upper gastrointestinal series.

In the first patient of the series readmitted with a leak, there were septic shock and massive pulmonary embolism, and the drainage of peritoneum was achieved by laparotomy. An attempt to close the gastric defect was also performed. This patient died because of sepsis and respiratory distress (global mortality 0.2%, fistula correlated mortality 16.67%) (Figure 1).

Two patients had clinical stability and intra-abdominal abscess and underwent CT guided percutaneous drainage.

**Figure 2:**
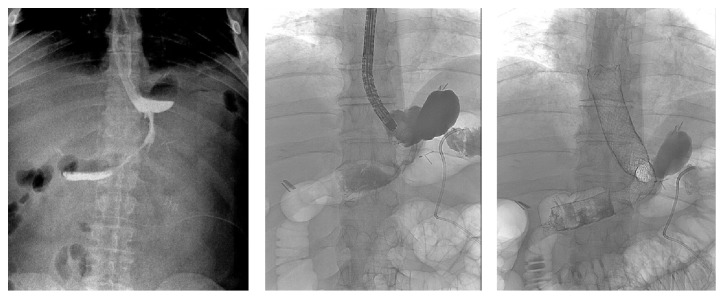
Male, 34 years, BMI 43 Kg/m^2^. Evidence of leak in radiological upper gastrointestinal series; confirmation of the leak with fluorescence endoscopy; endoscopic gastrooesophageal stenting.

After the resolution of the abscess, confirmed by CT, it was placed an endoscopic gastrooesophageal stent with complete resolution. One of these is shown in Figure 2.

Three patients were accepted to A&E in shock condition due to fistula; then they were treated by laparoscopic approach. The nasogastric tube was placed on arrival in A&E and removed early at the time of endoscopy and stent placement.

The reoperation was followed by endoscopic gastrooesophageal stenting.

In all cases the endoluminal stent was a covered self-expanding metal Beta™ Stent (Taewoong Medical) 20 cm long and 24 mm in diameter, because even though we are closest to the bougie, the gastric pouch has, also in consideration of the elasticity of the stomach, a larger diameter.

The proximal portion of the stent has been positioned in the oesophagus, while the distal portion was at the antrum-pyloric region.

When the diameter of the oesophagus was too wide and sufficient anchorage could not be obtained, we proceeded to the placement of a silk thread anchored to the stent and brought out of the nose.

The upper portion of the stent was positioned in the distal oesophagus and the inferior one in the stomach below the leak point. Of the 5 patients treated with stenting in 3 cases the stent was repositioned for minor displacements. No further postoperative complications were observed.

The mean duration of stenting was 15 days (range 14–16 days, SD ± 1).

The mean duration of TPN was 16 days (range 13–19 days, SD ± 2.83).

The mean time for resolution of the leaks was 55.5 days (range 26–83 days; SD ± 25.44).

## 4. Discussion

Literature data showed that sleeve gastrectomy is a safe and effective procedure.

Bariatric surgeons have not reached yet a general consensus about the best management of staple line leaks following LSG [5, 6].

Gastric staple line leak is the most important complication of LSG and can be life threatening, with prolonged intensive care unit hospitalization, reoperations, and even mortality [7].

Several conditions like ischemia, poor surgical technique, stapler failure, high intragastric pressure, and diathermy related organ injury can cause a leak after sleeve gastrectomy [8].

Current data shows a post-LSG incidence of leak ranging from 0.5% to 7% in different series [9].

Recently Gagner [10] reported that this incidence is decreasing from an initial generally accepted rate of 2.5% to 1.1% in 2013, as reported in a large cohort of 46.133 sleeve gastrectomies, with a decreased incidence of more than 50% [11].

Leaks can result in significant morbidity, with an associated mortality rate of 0.1–0.2% [12, 13].

Early diagnosis, management, and treatment of a gastric leak after LSG are difficult and still a matter of debate.

In our experience, all patients were discharged home in good clinical conditions, with the leak being diagnosed several days after surgery, as confirmed by Sethi et al. [14] in a report of 1762 LSGs.

The same authors demonstrated that the sensitivity of the routine postoperative radiological UGI series was 10.5% [95%; CI (1.3, 33.1)] and the specificity was 100% [95%; CI (98.2, 100)].

Our experience agrees with the results of previous studies, in which routine postoperative UGI series is not an ideal technique to screen all patients for leaks.

When the diagnosis of a gastric leak is made, it represents a challenge for the bariatric surgeon, who needs to decide among different approaches: conservative, percutaneous, or surgical exploration.

Szewczyk et al. [15] do not recommend attempting to suture the leak because this technique did not prove to be appropriate. Sutures are applied to tissues affected by a severe inflammatory process with low ability to maintain the margins of the leak closed and to obtain the healing.

If an exploratory laparoscopy is performed, it should be done in order to drain the area where the leak is from, wash out the infected fluid, and collect sample for bacterial cultures.

Endoscopic stenting after staple line leaks has been supported by many authors in recent years [16–18], even if this is not a widely accepted treatment.

Stent migration is the main complication after the procedure and it has been reported in 20%–59% of cases among different series [19–21].

In our experience, stents 20 cm long and 24 mm in diameter were chosen. In this way we obtained, in each case, a good adherence of the stent to the gastric wall.

The shape of the proximal part of the stent and its angle with respect to the stent body allowed complete coverage of the leak, thus promoting the healing process.

An uncontained leak, or one associated with hemodynamic instability, requires urgent operative intervention. Based on our experience, we suggest the following flow chart for the treatment of staple line acute and early leaks after LSG.

If there is clinical stability and no evidence of intra-abdominal abscess the patient should be treated with conservative treatment with fasting, total parenteral nutrition, intravenous antibiotic treatment, and gastrooesophageal stent placement.

If there is clinical stability and evidence of an intra-abdominal abscess this treatment should be preceded by a CT guided percutaneous drainage. If you do not have a biochemical response at 48 hours we proceed to exploratory laparoscopy.

On the other hand, if the patient comes to A&E with septic shock, the first treatment should be represented by a laparoscopic exploration with washout and drainage, in order to remove the infected collection, and when clinical stability is obtained the patient treatment can be completed by fasting, total parenteral nutrition, intravenous antibiotic treatment, and stent placement.

**Figure 3:**
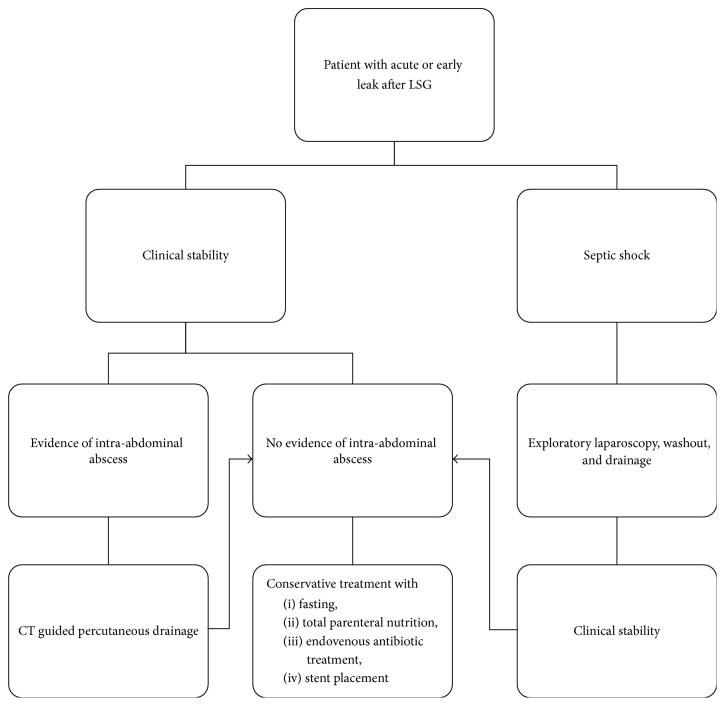
Flow chart for the management of gastric leaks.

The treatment protocol (Figure 3) was designed according to the international sleeve gastrectomy expert panel consensus statement [4].

## 5. Conclusion

Sleeve gastrectomy is an effective and relatively safe procedure for morbid obesity.

The treatment of patients who develop leakages requires a multidisciplinary team.

Choosing the proper treatment depends on clinical stability and on the presence or absence of abscess. Our treatment protocol showed being associated with low complication rate and minor discomfort to the patients, reducing the need for more invasive procedures.

In consideration of the small number of leakages, further studies, based on larger series of morbidly obese patients, are needed in order to validate this approach.
